# A Case Report and Review of the Literature of Penile Metastasis From Rectal Cancer

**DOI:** 10.3389/fsurg.2022.814832

**Published:** 2022-05-09

**Authors:** Azuolas Kaminskas, Ausvydas Patasius, Marius Kincius, Virginijus Sapoka, Rytis Zilevicius, Aušra Garnelytė, Audrius Dulskas

**Affiliations:** ^1^Department of Abdominal and General Surgery and Oncology, National Cancer Institute, Vilnius, Lithuania; ^2^Department of Oncourology, National Cancer Institute, Vilnius, Lithuania; ^3^Faculty of Medicine, Vilnius University, Vilnius, Lithuania; ^4^National Center of Pathology, Affiliate of Vilnius University Hospital Santaros Klinikos, Vilnius, Lithuania

**Keywords:** penile metastasis, rectal cancer, corpus spongiosum, case report, literature review

## Abstract

**Background:**

Metastatic involvement of the penis in cases of rectal cancer is exceptionally rare condition. Our clinical case report and review of the literature will contribute in complementing currently limited data on penile metastasis from rectal cancer.

**Case report:**

We report a case of a 64-year-old male diagnosed with penile metastasis from rectal cancer. The patient was treated with neoadjuvant chemoradiotherapy followed by total mesorectal excision (TME). However, penile metastasis developed 3 years later, clinically presenting as penile pain and solid formations along the entire length of the penis with visible tumor in the head of the penis. The amputation of penis was performed, and adjuvant chemotherapy was prescribed. The patient survived only 6 months.

**Conclusion:**

Penile metastasis from rectal cancer in most cases is a lethal pathology that indicates wide dissemination of oncological disease and has a very poor prognosis. Aggressive surgical treatment is doubtful in metastatic disease as this will negatively affect the quality of life.

## Introduction

Colorectal cancer is one of the most common oncological diseases worldwide, which ranked third in terms of cancer incidence in 2020 (1). Most often, rectal cancer metastasizes to the liver, lungs, bones, and the peritoneum (2). Other atypical sites of metastases of rectal cancer are singular, and metastatic involvement of the penis is exceptionally rare condition, about which there is a relatively little information in the literature. According to our search, until now, there are only 72 cases reported in the literature (Table 1).

**Table 1:** A literature review of previously published penile metastasis cases from rectal cancer.

**References**	**Age, years**	**Treatment of primary tumor**	**Penile MTS occurrence after treatment of primary tumor, months**	**Initial symptoms and signs**	**Treatment of penile MTS**	**Other sites of MTS**	**Survival, months**
Eberth (3)	40	–	–	0.5 cm bulb lesion	–	–	–
Cattell and Mace (4)	30	APR	29	Priapism, mass	Resection	–	9 years, alive
Bowersox and Frerichs (5)	68	Palliative	Penile metastasis was observed prior to primary cancer	Nodules	No treatment	Observed (diaphragm)	2.5
Poutasse (6)	51	APR	7	Nodule, urination difficulties	Amputation	Observed	12
Poutasse (6)	72	Palliative	2	Urination difficulties, nodules	Palliative (urethral catheter)	Observed (liver)	4
Boyd (7)	54	APR	48	Urination difficulties, mass near perineum, nodule	Amputation	–	11, alive
Oehlschlaegel (8)	–	–	–	–	–	–	–
Tagart (9)	75	APR	108	Hard swelling in the shaft of the penis	Amputation	–	–
Pond and Wade (10)	64	APR	5	Urination difficulties,mass	Partial penectomy	Observed (skull)	20 days
Poser and Kuttig (11)	–	–	–	–	–	–	–
Bachrach and Dahlen (12)	59	No treatment	10 days after finding primary tumor	Visible plaque-like area	No treatment	Observed (liver, regional lymph nodes)	14 days
Selikowitz and Olsson (13)	48	APR	60	Nodular induration of the penis, urination difficulties	Palliative treatment (cystostomy)	Observed (previous perineal wound site, pelvis)	6
Selikowitz and Olsson (13)	75	Palliative	6	Urination difficulties, nodular induration	Palliative (cystostomy, chemotherapy)	Observed (liver)	2
Rees (14)	41	APR	27	Urination difficulties, nodules	Palliative (chemotherapy, urethral catheter)	–	8, alive
Rees (14)	71	APR	36	Urination difficulties	Palliative (radiotherapy)	–	–
Baron and Pinck (15)	–	–	–	–	–	–	–
Kumar and Newland (16)	70	APR	4	Urination difficulties, nodes	Palliative (chemotherapy, irradiation to the penis)	Observed (liver)	5 days
Zanetti et al. (17)	–	–	–	–	–	–	–
Okumura et al. (18)	45	Hartmann's resection	22	Priapism	–	Observed (lungs)	–
Honda et al. (19)	60	APR	24	–	–	–	–
Khubchandani (20)	71	APR	40	Nodules	Chemoradiotherapy	Observed (lungs, pelvis)	19
Mukamel et al. (21)	58	–	2	Priapism	No treatment	–	5
Haddad and Manne (22)	67	Palliative	6	Nodules	–	–	3 weeks
Doré et al. (23)	58	–	–	–	–	–	–
Comandone et al. (24)	–	–	–	–	–	–	–
Ben-Yosef and Kapp (25)	58	APR, radiotherapy	5	–	Radiotherapy and hyperthermia	Observed (bones, liver)	3
Kupferet al. (26)	67	–	–	–	–	–	–
Cuvillieret al. (27)	–	APR	29	Nodules	Chemotherapy	–	15
Lange et al. (28)	42	–	–	–	–	–	–
Al-Mashat et al. (29)	65	APR	19	Nodule, dysuria	No treatment	Observed (perineum, rib)	5
Sukumar and Qureshi (30)	75	APR, chemotherapy, radiotherapy	2	Nodules, ulcers	No treatment	–	2
Tan et al. (31)	53	APR	At same time	Nodules	Chemoradiotherapy	–	–
Yilmaz et al. (32)	71	LAR	24	Penoscrotal urethral fistula, priapism	Chemotherapy	Observed (perineum, pelvis)	2.5
Lo and Crew (33)	56	APR, chemoradiotherapy	24	Nodules	Radiotherapy	–	–
Cathomas et al. (34)	58	Chemotherapy, LAR with TME	26	Nodules	Palliative (radiotherapy)	Observed (lungs)	–
Appu et al. (35)	65	APR	24	Nodules	Chemoradiotherapy	–	12
Laca et al. (36)	61	Surgery and chemotherapy	18	Priapism, lesions of the prepuce	Circumcision	–	–
Pellicé i Vilalta (37)	–	–	–	–	–	–	–
Cherian et al. (38)	73	APR, chemotherapy	60	Ulcero-proliferative lesions, penile discharge	No treatment	Observed (lungs)	4
Ketata et al. (39)	59	APR	312	Nodules	Chemotherapy	Observed (liver)	16, alive
Murhekar et al. (40)	78	APR	24	Urination difficulties, nodules	No treatment (refused)	–	4
Chung et al. (41)	69	–	At same time	Nodules	Chemoradiotherapy	Observed (liver)	6, alive
Küronya et al. (42)	65	LAR	54	Nodule	Chemoradiotherapy	–	–
Park et al. (43)	43	APR, chemoradiotherapy	24	Priapism, nodules	Radiotherapy	Observed (para-aortic lymph nodes, lungs, vertebra)	–
Yildirim et al. (44)	78	APR, chemoradiotherapy	24	Ulcerous lesions, urination difficulties	Chemotherapy	Observed (vertebrae, sacroiliac joint)	3
Madrigal-Medina (45)	45	Chemoradiotherapy, APR	Few months	Ulcers	Refused	Observed (liver, lungs)	1
Lee et al. (46)	54	APR	18	Nodules	Radiotherapy	–	–
Gbenou et al. (47)	79	Rectocolectomy	24	Papulonodules	Palliative	Observed (presacral masses)	6
Maestro et al. (48)	70	–	18	Indurations of the penis	–	–	–
Dorsett et al. (49)	60	Chemoradiotherapy	–	Mass	Penectomy	–	4
Kimura et al. (50)	57	Chemotherapy, total pelvic exenteration	9	Bloody discharge from the penis, nodule	Penectomy, chemotherapy	–	24, alive
McGuinness et al. (51)	61	Chemoradiotherapy, APR, chemotherapy	60	Lumps on glans penis	Radical circumcision, glansectomy, local radiotherapy	Observed (lungs, pelvis)	4
Persec et al. (52)	43	APR, chemoradiotherapy	24	Ulcerated nodular lesions, induration, urination difficulties	Local excision, palliative (cystostomy)	Observed (lungs, peritoneum)	6
Papaefthymiou et al. (53)	78	Chemoradiotherapy, APR	24	Nodule	Chemotherapy	–	3
Luo et al. (54)	54	–	–	–	Palliative (colostomy, chemotherapy)	Observed (liver)	10
Hajianfar et al. (55)	78	LAR, chemoradiotherapy	8	Nodules	Partial penectomy	–	–
Chang et al. (56)	73	LAR, chemotherapy	10	Urination difficulties, nodules	Resection, chemoradiotherapy	–	12, alive
Cholin et al. (57)	88	–	At same time	Lesion	No treatment	Observed (retrocrural, para-aortic, and parailiac lymph nodes, liver, adrenals, lungs)	9 weeks
Brønserud et al. (58)	–	Radiotherapy, APR	24	Palpable mass	–	Observed (lungs)	36
Alzayed et al. (59)	70	–	26	–	–	–	–
Delto et al. (60)	80	LAR, chemotherapy	24	Nodules	Chemotherapy	Pulmonary nodule	–
Nunes et al. (61)	66	APR	24	–	Radiotherapy, chemotherapy	–	–
Fabiani et al. (62)	78	Surgical resection, radiotherapy	60	Nodule	Chemotherapy	Recurrence of rectal cancer	17
Christodoulidou et al. (63)	70	Chemoradiotherapy, LAR, chemotherapy	24	Node	Penectomy	Observed (lungs)	–
Kozan et al. (64)	58	Chemoradiotherapy, APR, chemotherapy	18	Visible mass	Penectomy	–	Alive
Fuente et al. (65)	70	Chemotherapy, APR	30	Urination difficulties, induration, ulcers	Chemotherapy	Observed (pelvic bones)	12, alive
Efared et al. (66)	46	Chemotherapy, APR	8	Induration	Chemotherapy	–	Alive
Kuliavas et al. (67)	41	Radiotherapy, LAR, chemotherapy	17	Dysuria	Penectomy	–	2
Marghich et al. (68)	47	Chemoradiotherapy, APR, chemotherapy	4	Nodule	Chemotherapy	Observed (lungs, iliac, lombo-aortic, celio-mesenteric, and inguinal lymph nodes, bones)	4, alive
Lee et al. (69)	74	Chemoradiotherapy, APR, chemotherapy	9	Nodules	Chemotherapy	–	4, alive
Zang and Yang (70)	66	APR, chemotherapy	36	No signs and symptoms	Penectomy, chemotherapy	–	Alive
Our case	64	Chemoradiotherapy, TME	36	Tumor and solid infiltrations	Amputation, chemotherapy	–	6

*MTS, metastasis; APR, abdominoperineal resection; LAR, low anterior resection; TME, total mesorectal excision*.

Here, we present a case of the patient who developed penile metastasis from rectal cancer and review the existing literature.

## Case Description

A 64-year-old male came to our clinic complaining of blood presence in the stool. The patient underwent a lower gastrointestinal tract endoscopy, and rectal cancer 7 cm from the anal verge was detected and confirmed with biopsy (moderately differentiated adenocarcinoma). The patient then underwent chest and abdominal computed tomography (CT) scan with pelvic magnetic resonance imaging (MRI) – the clinical diagnosis of middle rectal cancer (cT3N1) was confirmed. The patient was treated with neoadjuvant chemoradiotherapy (consisted of 50·4 Gray (Gy) in 28 fractions of 1·8 Gy per day for 5½ weeks with continuous infusion of fluorouracil (1,000 mg/m2 per day for 5 days) during the 1st and 5th weeks of radiotherapy). Eight weeks following the neoadjuvant treatment, follow-up MRI was performed. No tumor was visible, and only single suspicious lymph node in the mesorectum was seen. Patient underwent total mesorectal excision (totally 12 weeks following the neoadjuvant chemoradiotherapy). Resected specimen was examined by pathologists, and the diagnosis of moderately differentiated (G2) rectal adenocarcinoma with metastases to regional lymph nodes (ypT2N1b 2 of 15 lymph nodes), R0, and complete TME was confirmed. The postoperative course was uneventful, and adjuvant chemotherapy was not prescribed.

**Figure 1:**
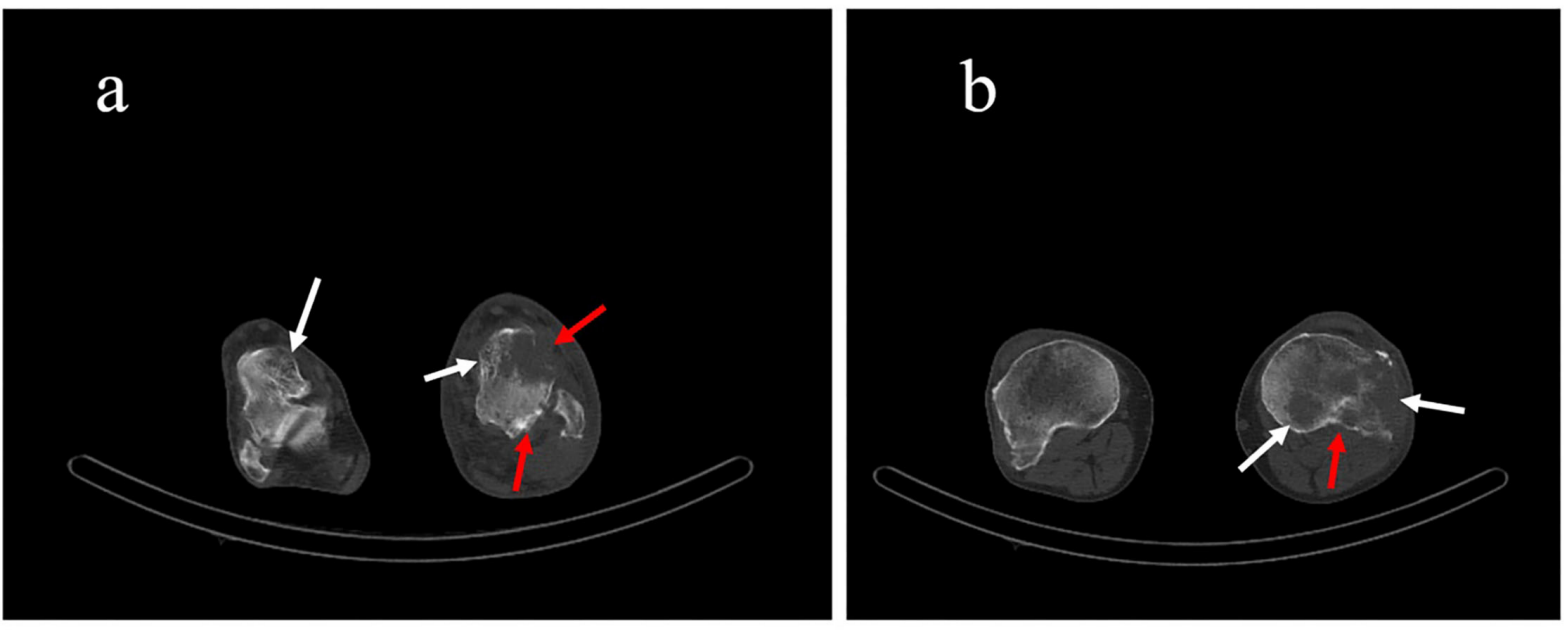
**(a)** Axial and **(b)** sagittal computed tomography scan planes, showing irregular accumulation of contrast in the penis without distant metastases (white arrows).

**Figure 2:**
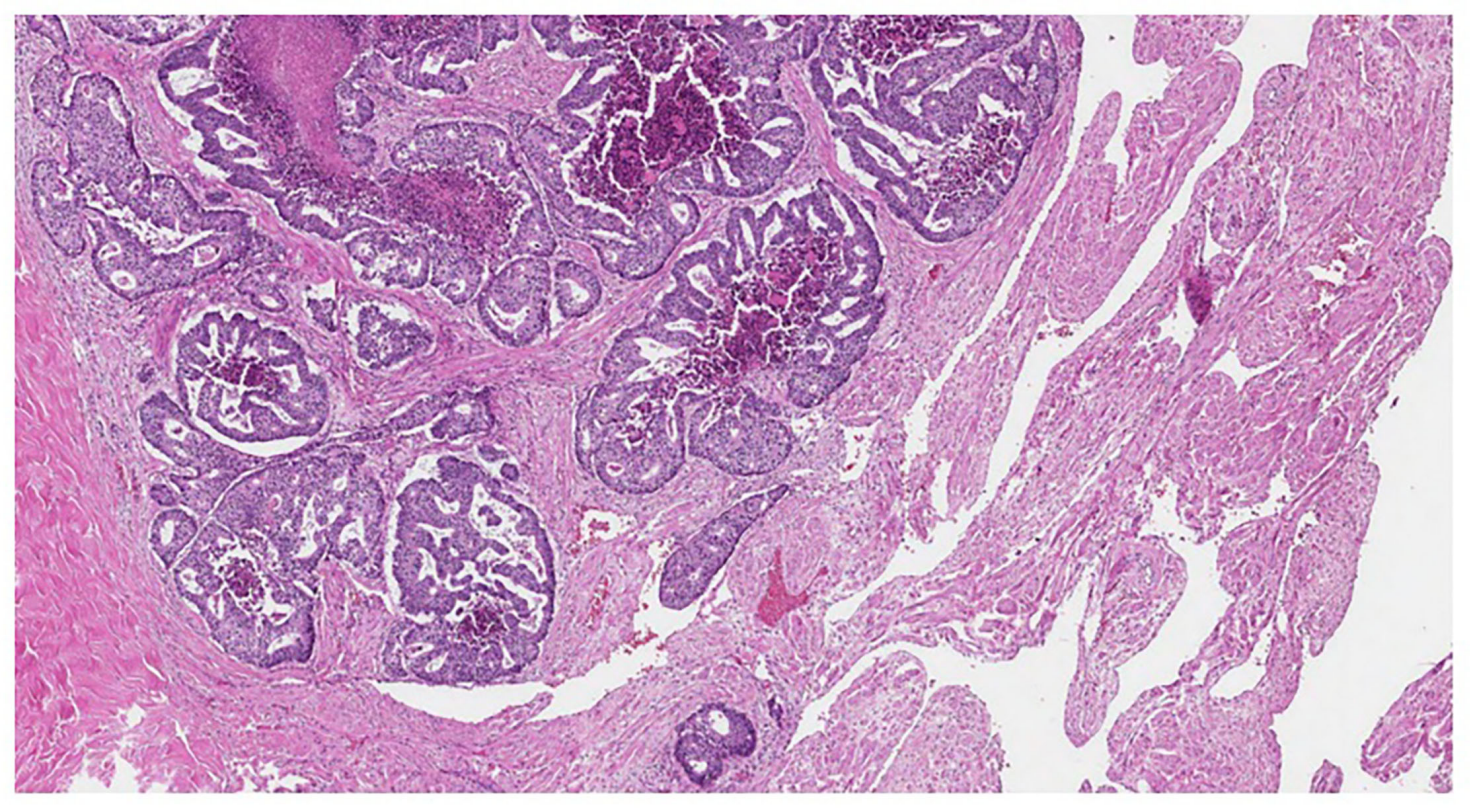
Cribriform tumor structures in the corpus cavernosum (HE, original magnification ×40).

**Figure 3:**
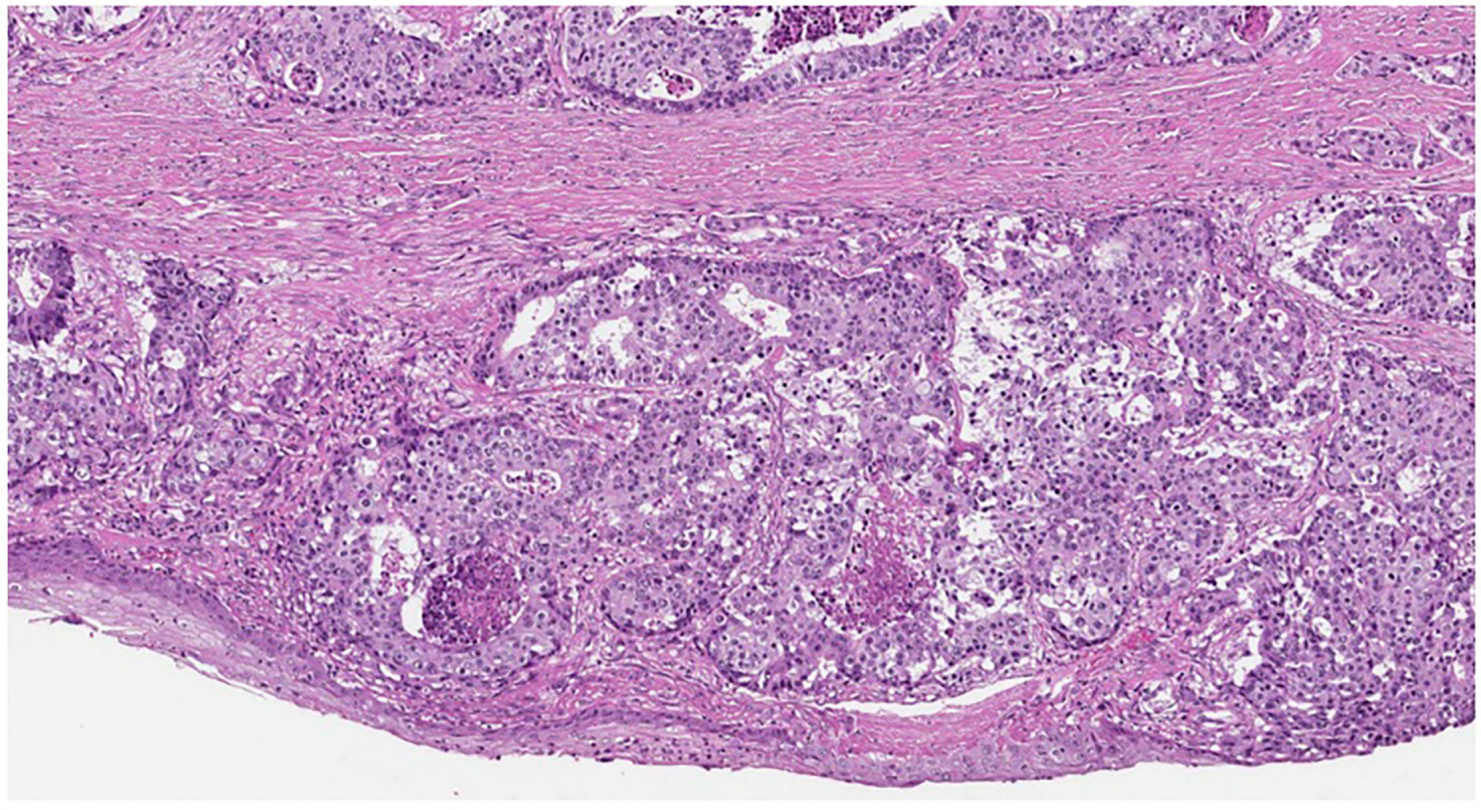
Tumor structures near the urethra (HE, original magnification ×100).

**Figure 4:**
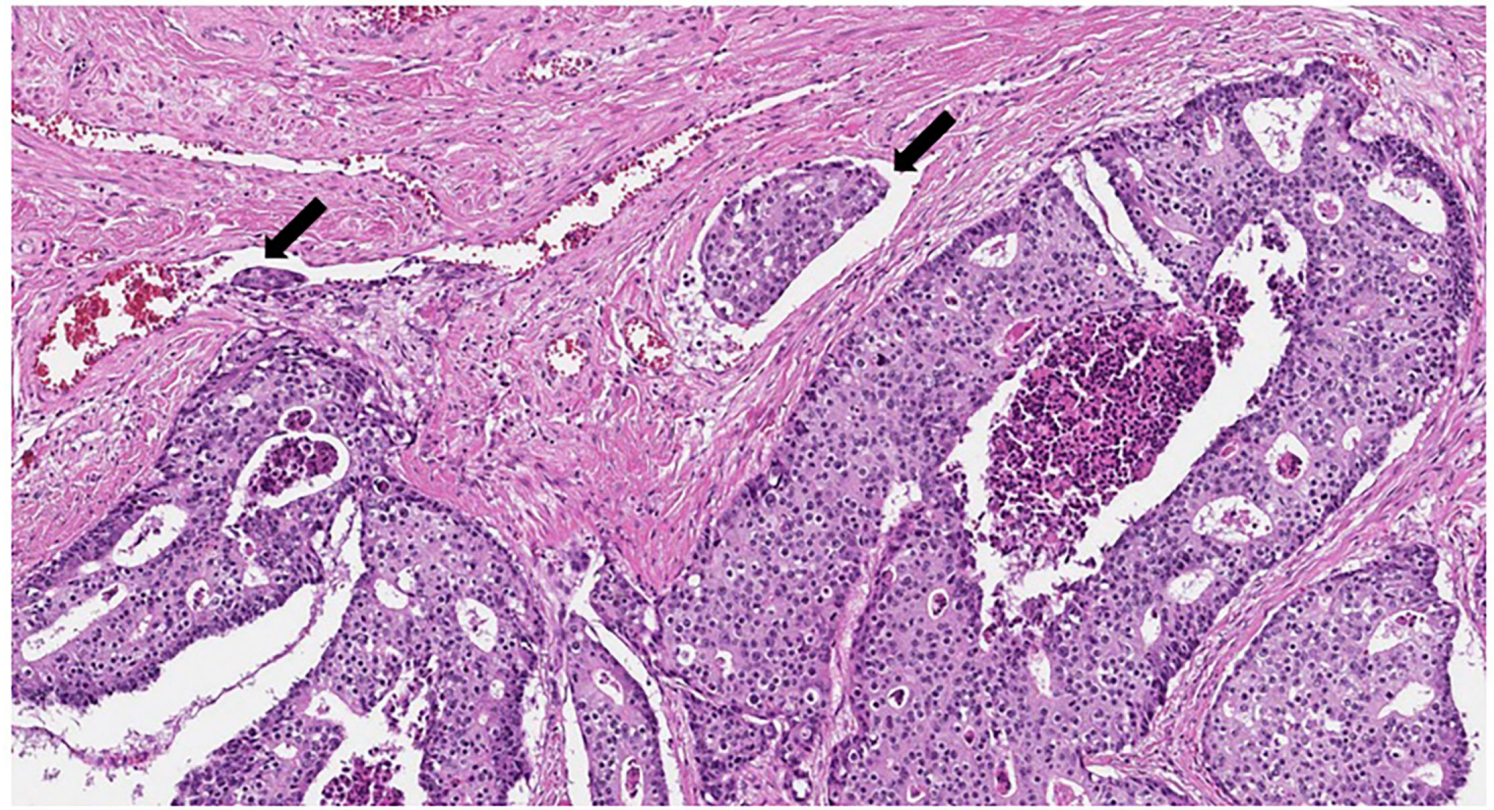
Cribriform tumor structures with dirty necrosis. Lymphovascular invasion (marked with arrows) (HE, original magnification ×100).

**Figure 5:**
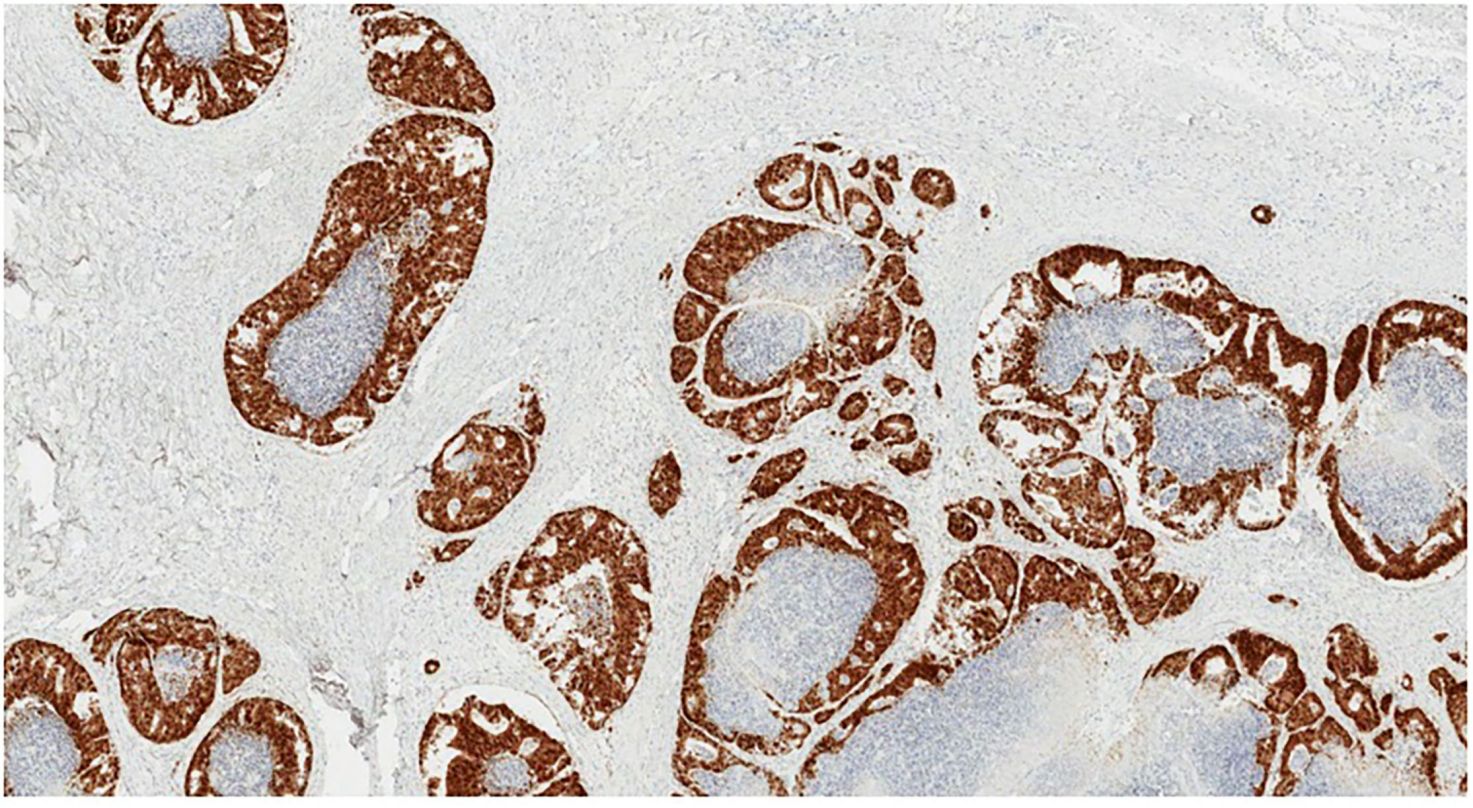
CDX2 immunohistochemistry. Positive nuclear staining in tumor cells (original magnification ×40).

**Figure 6:**
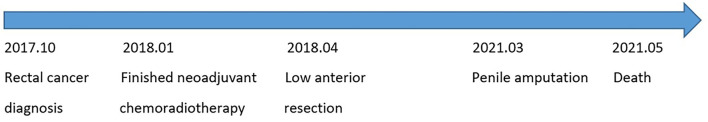
Timeline of the presented case.

Three years later, the patient started complaining of penile pain and solid formations along the entire length of the penis. During physical examination, a rough and raised tumor with unclear boundaries was observed in the head of the penis, and solid infiltrations were observed in the corpora cavernosa, extending all the way to the root of the penis. The patient underwent a CT scan, which showed irregular accumulation of contrast in the penis without distant metastases (Figure 1). The patient was discussed by a multidisciplinary team, and it was decided to treat the patient by performing an amputation of the penis. Pathological examination of the resected specimen revealed the penile metastasis of low-grade (G2) adenocarcinoma of the colon with lymphovascular invasion and visible tumor structures in the resection margins (R1). Histologically, tumor was composed of irregular glandular and cribriform structures lined with columnar cells with eosinophilic cytoplasm and polymorphic-stratified nuclei. The tumor contained abundant necrotic debris (“dirty necrosis”) in the lumen of the structures. The tumor was located in penile glans and corpus, spreading to both corpus spongiosum and cavernosum, infiltrating surrounding adipose and striated muscle tissues. Perineural and lymphovascular invasions were observed as well. Immunohistochemically, the tumor cells were positive for CDX2, which is a marker of the intestinal epithelium and helps to determine the primary location of metastatic colorectal adenocarcinomas (Figures 2–5). The patient was scheduled for adjuvant chemotherapy with XELOX regimen with a reduced dose of Capecitabine because of the DPYD gene polymorphism heterozygous variant. Almost 3 months after the operation, hematuria occurred, and then suprapubic cystostomy was performed. Later, the patient complained of severe pain in his right leg, and x-ray with bone scintigraphy imaging tests showed osteolytic-type metastases, and massive bone damage with tumor masses in both legs (Figure 2). Due to the progression of the disease while on chemotherapy and deteriorating general condition of the patient, it was decided to apply symptomatic (palliative) treatment. The patient died 3 months later (see the timeline in Figure 6).

## Discussion

Here, we present a rare case of rectal cancer metastasis to penis with fast deterioration and an unfavorable ending.

The location of the penis is closely surrounded by other anatomical structures, which are often the sites of origin of primary tumors. Moreover, sufficient blood flow of the penis is ensured by an abundant amount of vascular structures (71). However, despite these factors, metastatic involvement of the penis is extremely rare (14, 65, 72, 73). So far, only 72 cases have been described in the literature (Table 1). The most common sites of origin include the bladder, prostate, rectum (as in our and previous cases), and kidneys (65, 73, 74). To this day, it has not been entirely clear how primary tumors metastasize to the penis. The literature indicates several possible ways of primary tumors spread to the penis (73, 75, 76), such as direct extension, retrograde venous metastasis, retrograde lymphatic metastasis, direct extension into arterial pathways, spread by instrumentation, secondary, tertiary or paradoxical embolism (the retrograde venous mechanism is considered as the main pathway of tumor spread to the penis) (73, 75, 76). In our case, it is not entirely clear which of these mechanisms played the main role. We consider that retrograde venous or lymphatic mechanisms are most likely because the patient was diagnosed with rectal adenocarcinoma metastasing to the regional lymph nodes(ypT2N1b).

According to our review, the mean patients' age presenting with penile metastases is 62 years. Moreover, our findings are consistent with data from other studies, indicating that, on average, penile metastases occur 2–3 years (in our findings – 27 months), following the treatment of the primary tumors (27, 67). Clinical presentation consists of nodules, masses or induration of the penis, lesions/ulcerations, priapism, urination problems, penile or perineal pain (67, 73, 75–77). We found that the most common initial signs and symptoms were nodules and urination difficulties. The diagnosis of penile metastasis can be confirmed histologically by performing biopsy or fine-needle aspiration (73). Other noninvasive diagnostic tests, such as ultrasound scan, magnetic resonance imaging or computed tomography scan, could be informative and can help to detail local margins of the tumor as well as to visualize systemic dissemination of the disease (73). In our case, the patient was 64 years old, and was presented with penile pain, solid formations, and rough and raised tumor 3 years after the treatment of rectal cancer. We used CT scan with contrast to detail the possible spread of the tumor. We did not perform pelvic MRI because of the degree of presented symptoms. The patient needed urgent surgery for pain management and urinary dysfunction.

As this entity is very rare, there are no international or national treatment guidelines. Possible treatment modalities alone or in combinations include local excision of the tumor, total penectomy, chemotherapy, radiotherapy, and palliative treatment only (14, 39, 50, 67, 69). In our review, chemotherapy (alone or in combination with radiotherapy or surgical treatment) was the most often suggested treatment. Other studies suggest that radical surgical treatment could be the best option for patient survival improvement. However, the current data are lacking (14, 22, 67). Most commonly, penile metastases accompany systemic spread, and only palliative treatment is possible to improve the patient's quality of life. One should keep in mind that radical surgical treatment will definitely worsen the quality of life (38, 67). In our case, we initially treated the patient by performing total penectomy and prescribing adjuvant chemotherapy as he presented with painful masses and urinary dysfunction. However, the disease progressed instantly, and the patient then was scheduled for palliative care only.

Despite possible radical treatment modalities, such as total penectomy, the prognosis of patients with penile metastases is poor. Usually, penile metastases indicate widespread oncological disease, and the survival of these patients ranges from a few to several months only (4, 14, 27, 38, 67). Of the 72 cases reviewed, 31 patients had systemic spread of the disease at the time of the diagnosis of penile metastasis. It is important to note that not all authors provided information on the systemic spread of the disease, so the actual number of cases with disseminated pathology may be higher. The average survival from the time of the diagnosis of penile metastasis was about 9 months. Our patient developed metastases to the bones almost 3 months after total penectomy, and the overall survival of the patient was 6 months following amputation of penis.

To conclude, penile metastases from rectal cancer are extremely rare, indicating wide dissemination of the oncological process with a very poor prognosis. Aggressive surgical treatment is doubtful in metastatic disease as this will negatively affect the quality of the patient's life.

## Data Availability Statement

The raw data supporting the conclusions of this article will be made available by the authors, without undue reservation.

## Ethics Statement

The studies involving human participants were reviewed and approved by National Cancer Institute Review Board. The patients/participants provided their written informed consent to participate in this study.

## Author Contributions

AD and AP conceived the idea. AK and AD wrote the article draft. RZ, MK, VS, AD, and AK performed the literature search. VS performed the analysis of the data. All authors contributed to the article and approved the submitted version.

## Conflict of Interest

The authors declare that the research was conducted in the absence of any commercial or financial relationships that could be construed as a potential conflict of interest.

## Publisher's Note

All claims expressed in this article are solely those of the authors and do not necessarily represent those of their affiliated organizations, or those of the publisher, the editors and the reviewers. Any product that may be evaluated in this article, or claim that may be made by its manufacturer, is not guaranteed or endorsed by the publisher.
